# Recent Anti-platelet Therapy Revealing Underlying Undiagnosed Gastrointestinal Stromal Tumor in Otherwise Healthy Patient

**DOI:** 10.7759/cureus.7450

**Published:** 2020-03-28

**Authors:** Shehriyar Mehershahi, Haider Ghazanfar, Danial H Shaikh, Ahmed Baiomi, Ariyo Ihimoyan

**Affiliations:** 1 Gastroenterology, BronxCare Health System, Bronx, USA; 2 Internal Medicine, BronxCare Health System, Bronx, USA

**Keywords:** gist, aspirin, upper gastrointestinal bleed

## Abstract

Gastrointestinal stromal tumors (GIST) are uncommon tumors of the gastrointestinal (GI) tract that arise from primitive mesenchymal cells. Usually, GIST presents as chronic gastrointestinal symptoms or obscure gastrointestinal bleed. Not many cases have been reported in the literature with acute symptoms in the setting of recent aspirin use. We report a case of 70-year-old male otherwise healthy who presented to the hospital with the complaint of black tarry stool for the past two days after taking one tablet of aspirin once a day for two days. The patient underwent upper endoscopy which showed a moderate size polypoid mass in the gastric fundus. Initially, standard endoscopic biopsy was negative for malignancy, due to high suspicion for GIST, later the patient underwent upper endoscopic ultrasound with fine-needle aspiration which confirmed low-grade GIST.

## Introduction

Gastrointestinal stromal tumors (GISTs) are a group of stromal or mesenchymal neoplasms affecting the gastrointestinal tumors. Although GISTs are relatively uncommon (<1% of all gastrointestinal neoplasms) compared with adenocarcinoma, they are the most prevalent mesenchymal tumors of the gastrointestinal tract [1]. GIST is distinguished from myogenic or neurogenic tumors due to the expression and mutations of KIT in the c-kit. Mutation in the c-kit gene is associated with aggressive features and poor prognosis [2]. The stomach and small intestine are the most common locations [3]. GISTs are known to grow from intestinal cells of Cajal due to mutation of KIT and platelet-derived growth factor receptor alpha (PDGFRA) [4,5]. The diagnosis of gastric GISTs is often delayed because these tumors cause no characteristic symptoms. We present an acute obscure GI bleed with underlying undiagnosed GIST in a patient taking two tablets of aspirin for shoulder pain.

## Case presentation

Our patient is a 70-year-old male who presented to the hospital with the complaint of generalized weakness, feeling dizzy and black tarry stool for the past two days. The patient states that he took one tablet of aspirin once a day for two days for shoulder pain prior to the start of the black tarry stool. The patient denies any previous similar episode, hematochezia, weight loss, loss of appetite or constitutional symptoms. Past medical history is significant for hypertension and hyperlipidemia well controlled without medication. The patient’s past surgical history is significant for appendectomy in 2016. No significant family history of gastrointestinal malignancy. The patient smokes 2-3 cigarettes a day for the past 30 years, drinks five to six beers on the weekends, denies using any illicit substance.

**Figure 1:**
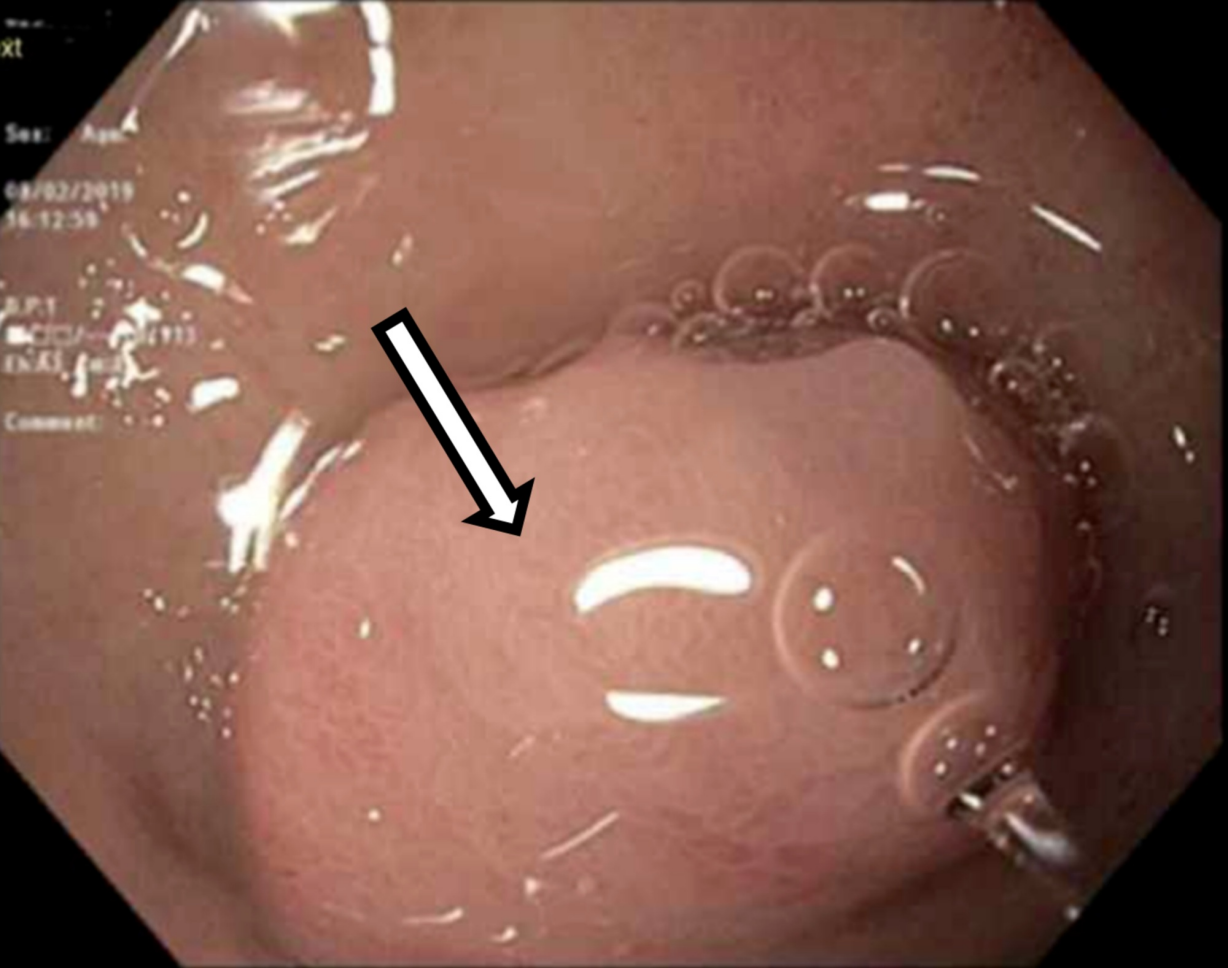
Esophagogastroduodenoscopy

**Figure 2:**
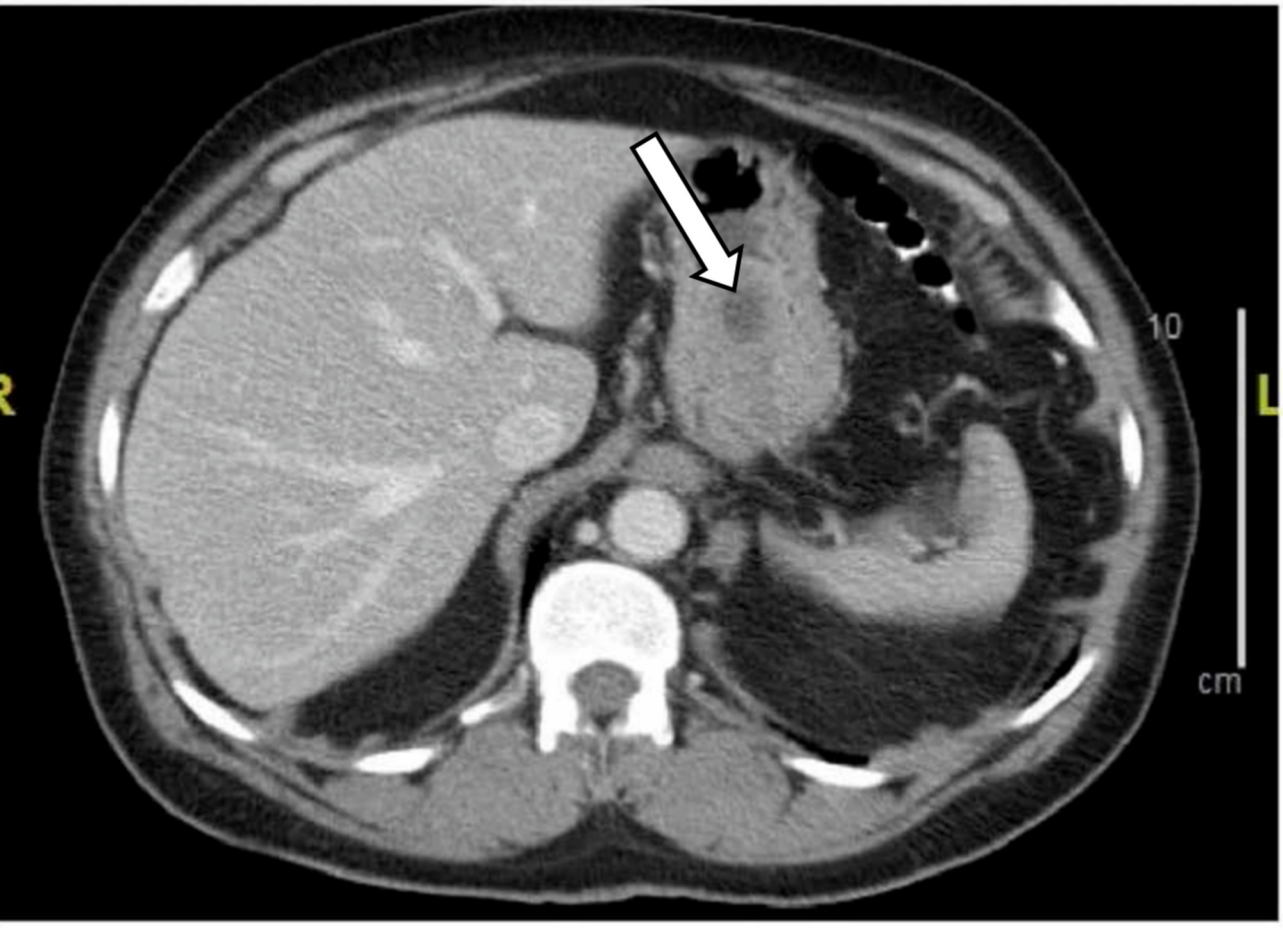
CT abdomen and pelvis with contrast material

In the emergency department the patient was found to be afebrile and had a blood pressure of 119/72 mmHg with the pulse rate of 81 bpm. On physical exam the patient was not in distress, the abdomen was soft, non-tender, non-distended. The digital rectal examination showed no hemorrhoids or hematochezia, finger staining with melanotic stool, no hematochezia. The rest of the physical examination was unremarkable. The patient was found to have hemoglobin of 8.3 g/dL on the presentation. The patient's baseline hemoglobin was 14.1 g/dl. On anemia workup the patient was found to have normal serum ferritin, folate, Vitamin B12, and iron serum levels. The patient underwent upper endoscopy which showed a moderate size polypoid mass in the gastric fundus with no ulceration or stigmata of bleeding. This has been shown in Figure 1. The biopsy revealed gastric antral mucosa with focal mild chronic inflammation and foveolar reactive changes. The patient underwent computed tomography (CT) abdomen and pelvis with contrast material which showed gastric mass 3.8 x 2.7 cm without evidence of metastatic disease to the chest, abdomen or pelvis. This has been shown in Figure 2.

**Figure 3:**
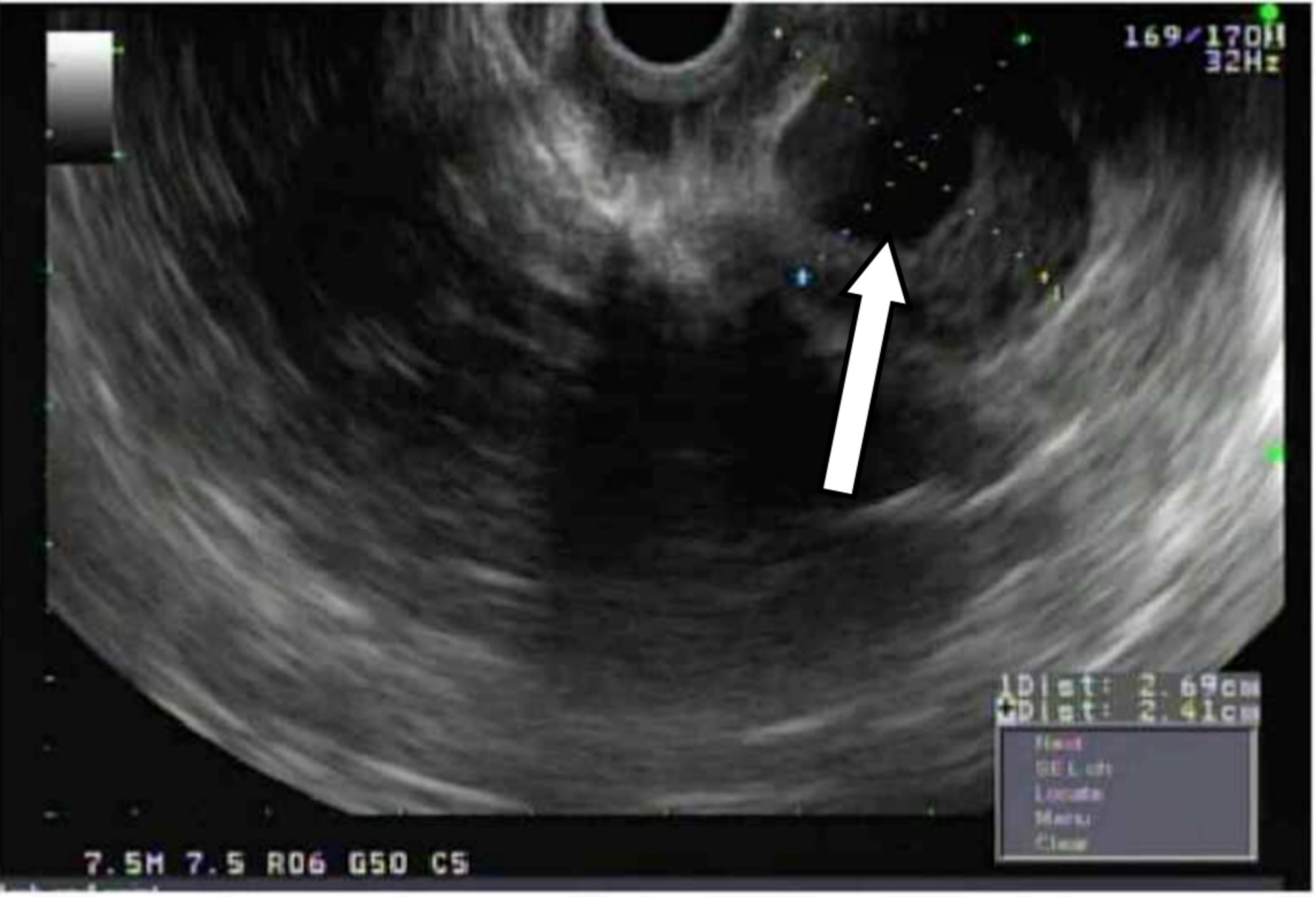
Endoscopic ultrasound

The patient underwent upper endoscopic ultrasound which showed oval intramural (subepithelial) lesion, located in the fundus 2.5 x 3 cm with a central cystic area. The lesion appeared to originate from within the muscularis propria (layer 4). This has been shown in Figure 3. Fine needle aspiration (FNA) was obtained for pathology. The pathology of the sample showed low-grade gastrointestinal stromal tumors (GIST). On immunohistochemical stain, the tumor cells were found to be positive for CD117, DOG1, and CD34, and it was negative for SMA antibodies. The patient underwent exploratory laparotomy and partial gastrectomy. Pathology tissue analysis confirmed gastrointestinal stromal tumor, spindle cell type measured 3.5 cm in greatest dimension, mitotic rate 7/5 mm^2^, high grade, and no lymphovascular invasion was seen. The patient had no complications postoperatively. The patient refused treatment with imatinib as he was scared of side effects.

## Discussion

The majority of the GISTs are identified clinically due to chronic and recurrent symptoms like GI bleeding or abdominal symptoms. Incidental findings during imaging or surgery account for up to 21% [6,7]. The most common site of GIST is the gastric fundus (55%) followed by corpus (27.6%) [8]. There is an estimated incidence of 0.3% to incidentally find subepithelial lesions during upper endoscopies [9]. It is difficult to distinguish between intramural and extramural tumors with traditional endoscopy alone. Most of the time the sample obtained from traditional endoscopic forceps biopsy is insufficient. The initial detection rate of traditional endoscopic forceps biopsy is 29% [10].

The traditional biopsy methods like excavating biopsy, snaring biopsy and endoscopic mucosal resection (EMR), are considered risky associated with frequent complications such as hemorrhage and perforation [11]. The advent of endoscopic ultrasonography (EUS) has resulted in a breakthrough by identifying the thickness of the wall, the layers of origin and guided tissue biopsy for diagnosing gastric GIST [12]. Al-Haddad et al. reported that the sensitivity, specificity and accuracy rates of EUS-FNA in the diagnosis of lymphoma were 80%-87%, 92%-93%, and 83%-89%, respectively [13].

Gastrointestinal bleeding caused by GIST is multifactorial and can be a result of altered local mucosal blood supply, cell necrosis or tumor invading and eroding the mucosal or submucosal blood vessels [14]. Liu et al. reported incidence rates of 26.4% and 54.7%, with gastric bleeding vs nongastric bleeding, respectively [15]. The stomach is larger and has more resistance to extrusion by GIST making it less prone to bleed than other non-gastric lesions. Patients with underlying un-diagnosed GIST on chronic antiplatelet therapy can initially present as obscure or overt GI bleeding [16].

Tumor size, mitotic rate, patient age and the location of the tumor are the independent prognostic factors [17]. According to the Armed Forces Institute of Pathology (AFIP) study, the overall survival was best in tumors confined to the esophagus and was worst in the tumors originating from the small bowel [18]. Tumor rupture has a significant negative impact on disease-free period and is associated with poor prognosis [19].

Surgical resection is the choice of treatment for all localized gastric GIST which are greater than 2 cm in size [16]. There are no clear guidelines regarding the management of GIST, which are less than 2 cm in size. As per Canadian guideline, GIST even less than 1 cm can be resected because of the risk of metastasis [20].

Our patient presented with the complaint of black tarry stools after taking aspirin for two days. There are few reports of upper GI bleed with underlying GIST as the culprit due to subsequent use of Clopidogrel and aspirin after recent drug-eluting coronary stent implantation [16]. It is very important to have a high clinical suspicion of underlying gastric neoplasm like GIST in patients taking non-steroidal anti-inflammatory drugs (NSAIDs), as the initial preliminary differential diagnosis of NSAIDs-induced gastric ulcer or erosion can lead to delay in diagnosis and treatment of these patients.

This case reinforces to prompt physicians to think of GIST as a differential in the acute presentation of upper GI bleed in the setting of recent aspirin use in an otherwise healthy patient. Patients with un-diagnosed GIST usually present with obscure or overt GI bleeding while on chronic antiplatelet therapy [16]. In our case the patient took two aspirin for two days and presented with an obscure GI bleed.

## Conclusions

Chronic antiplatelet therapy in the setting of underlying undiagnosed GIST can manifest with obscure or overt gastrointestinal bleed. Not many cases have been reported in the literature with early upper GI bleed in the setting of recent aspirin use in a patient with underlying un-diagnosed GIST. This case reinforces to prompt physicians to think of GIST as a differential in the acute presentation of upper GI bleed in the setting of two tablets of baby aspirin use in an otherwise healthy patient. Early diagnosis and prompt treatment are essential in treating GISTs.
